# Association of Treatment With Antipsychotics, Antidepressants, or Both With Movement Disorders and Seizures Among Children and Adolescents With Depression in Korea

**DOI:** 10.1001/jamanetworkopen.2022.7074

**Published:** 2022-04-15

**Authors:** Soo Min Jeon, Hae-Young Park, Susan Park, Un Sun Chung, Jin-Won Kwon

**Affiliations:** 1 BK21 FOUR Community-Based Intelligent Novel Drug Discovery Education Unit, College of Pharmacy and Research Institute of Pharmaceutical Sciences, Kyungpook National University, Daegu, South Korea; 2 Red Cross College of Nursing, Chung-Ang University, Seoul, South Korea; 3 Department of Psychiatry, School of Medicine, Kyungpook National University, and Department of Psychiatry, Kyungpook National University Children's Hospital

## Abstract

**Importance:**

Little evidence exists on neurological adverse events (movement disorders and seizures) that occur during adjuvant use of antipsychotics with antidepressants, especially in children and adolescents.

**Objective:**

To assess the association between neurological adverse events (movement disorders, including parkinsonism, dystonia, extrapyramidal symptoms, chorea, and tic, and seizures) and the adjuvant use of antipsychotics in children and adolescents with depression.

**Design, Setting, and Participants:**

A retrospective cohort study using the Health Insurance Review and Assessment claims database in Korea between 2008 and 2018. The study population was children and adolescents aged 2 to 18 years with depression who began treatment with antidepressants between January 1, 2010, and December 31, 2018. Data were analyzed between December 9, 2020, and December 10, 2021.

**Exposure:**

Time-varying exposure to antidepressants, antipsychotics, and concomitant use of antidepressants and antipsychotics. Concomitant use was further subdivided according to the antipsychotic treatment status (dose and agent).

**Main Outcomes and Measures:**

The extended Cox proportional hazards regression model, with adjustment for sex, age, health insurance type, psychiatric comorbidities, psychiatric hospitalization, and comedication with other psychotropic drugs, was used to estimate adjusted hazard ratios (aHRs) and 95% CIs for the associations of movement disorders and seizures with use of antidepressants and antipsychotics.

**Results:**

A total of 9890 patients were included in the study: 9541 (mean [SD] age, 14.8 [2.8] years; 4956 [51.9%] female) and 7731 (mean [SD] age, 14.9 [2.7] years; 4150 [53.7%] female) met the inclusion criteria for movement disorders and seizures, respectively. For movement disorders, associations were found between concomitant use (aHR, 3.68; 95% CI, 3.06-4.44) and antipsychotic-only use (aHR, 3.84; 95% CI, 3.03-4.87) compared with antidepressant-only use, but their CIs overlapped. The associations with seizure were similar (concomitant use: aHR, 2.06; 95% CI, 1.66-2.55; antipsychotic-only use: aHR, 2.05; 95% CI, 1.53-2.75). With concomitant use, the aHRs gradually increased with increasing doses of antipsychotics. Haloperidol had the highest aHR, 7.15 (95% CI, 3.89-10.00) for movement disorders. The highest aHR for seizure was observed with quetiapine (aHR, 2.36; 95% CI, 1.55-3.59), followed by aripiprazole (aHR, 2.05; 95% CI, 1.52-2.77).

**Conclusions and Relevance:**

In this cohort study, adjunctive antipsychotics with antidepressants were associated with movement disorders and seizures compared with antidepressant monotherapy in children and adolescents with depression. These results suggest that careful consideration of the risk-benefit profile of the antipsychotic use as adjuvant therapy in this population is needed.

## Key Points

**Question:**

Is treatment with antipsychotics in children and adolescents who are taking antidepressant medications associated with neurological adverse events?

**Findings:**

In this cohort study of 9890 children and adolescents prescribed antidepressants for depression, the hazard ratios of movement disorders and seizures with adjuvant antipsychotics were 2- to 3-fold higher compared with antidepressant monotherapy. Hazard ratios increased with increasing doses of adjuvant antipsychotics and varied according to which antipsychotic was concomitantly used.

**Meaning:**

These results suggest that adjuvant use of antipsychotics needs to be carefully considered in the treatment children and adolescents with depression.

## Teaser

This cohort study of data from the Health Insurance Review and Assessment database in South Korea assesses the associations between adjunctive therapy with antipsychotics and neurological adverse events, including movement disorders and seizures, in children and adolescents who were treated with antidepressants for depression.

## Introduction

Over the decades, multiple effective antidepressants have been developed to treat depression^1,2^; however, a significant proportion of patients generally do not achieve complete response and remission.^3,4,5,6^ Antipsychotics are commonly used as adjunctive therapy in adults.^6,7,8^ To date, the US Food and Drug Administration has approved 4 atypical antipsychotics (aripiprazole, quetiapine, brexpiprazole, and olanzapine) for this purpose.^9^ However, safety concerns still remain. In a meta-analysis of 16 randomized clinical trials (RCTs) of adults with treatment-resistant depression, augmentation with antipsychotics was associated with a higher discontinuation rate and more adverse events (AEs) than antidepressant monotherapy.^10^ Given that most RCTs have been conducted under strict inclusion criteria, short-term duration, and small sample size for rare events,^11,12,13^ the results from RCTs may be associated with more AEs or greater discontinuation rates than are seen in actual clinical practice.

Recently, the treatment of depression with antipsychotics has expanded to include children and adolescents as off-label therapy based on experience rather than evidence,^14,15,16,17^ which has raised concerns regarding their safety. In some studies, children and adolescents were more susceptible to some AEs, including extrapyramidal adverse effects, suggesting that these drugs might have different safety profiles in this population from those in adults.^16,18,19,20^ In previous studies conducted by some of us and other members of our research team, neurological AEs associated with antipsychotics (ie, movement disorder and seizure) were investigated in children and adolescents with psychotic disease using case-control^21^ and cohort designs.^22^ In the cohort study,^22^ the hazard ratio (HR) for movement disorder during a period of antipsychotic use vs a period of nonuse was 8.17 (95% CI, 7.16-9.33) and for the development of seizure was 3.47 (95% CI, 2.99-4.03); furthermore, higher doses of antipsychotic polypharmacy were associated with increased risk of both AEs, and individual agents had slightly different safety profiles. Both study designs showed robust results,^21,22^ indicating the need for careful monitoring and management of antipsychotic treatment in pediatric patients with psychotic disease regarding neurological AEs such as movement disorders and/or seizures.

To our knowledge, despite the increased use of antipsychotics in children and adolescents with depression, no studies have investigated the risk of the adjunctive use of antipsychotics in comparison with antidepressant-only use. Therefore, we aimed to investigate the association of movement disorders and seizures with the adjunctive use of antipsychotics for depression treatment in children and adolescents. In addition, we assessed the associations according to the coadministered antipsychotic treatment status, such as the dose and individual agent used.

## Methods

### Data Source

The Health Insurance Review and Assessment (HIRA) database of South Korea was used to conduct this retrospective cohort study. The HIRA provides anonymized data at the request of researchers after study proposals have been reviewed. South Korea has a universal single-payer health insurance system, the National Health Insurance Service, covering approximately 97% of the population. The remaining 3% of patients are under the Medical Aid Program, provided by the government for low-income individuals. The HIRA is an independent agency that reviews the claims data of patients under the National Health Insurance Service and Medical Aid Program from all health care organizations and medical facilities. Therefore, the HIRA database retains all medical records of the entire Korean population (approximately 50 million people). This database included the anonymized patient identification numbers, sex, age, type of insurance, *International Statistical Classification of Diseases and Related Health Problems, Tenth Revision* (*ICD-10*) diagnosis codes, and prescription records (active agents, prescription dates, duration, dosage, and administration routes). This study was approved by the Institutional Review Board of Kyungpook National University and was deemed exempt from the need for informed consent because of the use of deidentified data. The study followed the Strengthening the Reporting of Observational Studies in Epidemiology (STROBE) reporting guideline.^23^

### Study Population

This cohort study used the HIRA claims data from January 1, 2008, to December 31, 2018, on patients aged 2 to 18 years who had a history of antidepressant prescriptions after diagnosis with depression (*ICD-10* codes F32 and F33). The data analysis was performed between December 9, 2020, and December 10, 2021. We excluded patients who had any prescription record of antidepressants between 2008 and 2009 to include only incident cases. The index date was defined as the first prescription date of antidepressants. To construct a restricted cohort of antipsychotics initiators only for depression, we excluded patients who were prescribed antipsychotics or diagnosed with schizophrenia (*ICD-10* codes F20-F29), bipolar disorder (*ICD-10* code F31), manic episode (*ICD-10* code F30), intellectual disabilities (*ICD-10* codes F70-F79), and autism spectrum disorder (*ICD-10* code F84) within 2 years before the index date. In this way, the final study cohort was obtained, and we further divided the cohort into 2 overlapping cohorts, one group of participants who were free of movement disorders at baseline (the movement disorder cohort) and another group who were seizure free at baseline (the seizure cohort), by excluding patients with any history of diagnosis or medication prescriptions associated with each outcome within 2 years before the index date. In the movement disorder cohort, we excluded patients with any record of movement disorder diagnosis and prescriptions of antiparkinson or anticholinergic drugs before the index date. Patients who had diagnosis record of seizures or any prescription record of antiepileptics before the index date were excluded in the seizure cohort. eTables 1 and 2 in the Supplement summarize the diagnosis codes of our outcomes of interest and drug codes.

We compared the incidence of movement disorders and seizures in 4 time-varying exposures (antidepressant-only use, antipsychotic-only use, concomitant use, and no use) using a specific cohort per outcome. In each cohort, patients were followed up from the index date to the first date of the respective AE (movement disorder or seizure), the end of the 18th year of age, or the end of data collection (December 31, 2018), whichever occurred first.

### Drug Exposure

Exposure to antidepressants was determined according to Anatomical Therapeutic Chemical (ATC) classification system code N06A; exposure to antipsychotics was determined according to ATC code N05A (excluding lithium).^24^ For each cohort, we estimated the exposure of antidepressants and/or antipsychotics as time-varying covariates to explain changes in prescription patterns during follow-up. Patient drug use was classified into 4 categories: nonuse, antidepressant-only use, antipsychotic-only use, and concomitant use. Antidepressant-only use and antipsychotic-only use were defined as the person-time during which only drugs in one of the classes were available. If both drug classes were used simultaneously, we defined that person-time as concomitant use. An additional 14-day carryover duration following the end date of all prescriptions was considered in this process. Nonuse was defined as no prescriptions for the medications of interest.

Concomitant use was classified according to the average daily dose and individual antipsychotic agents. An average daily dose of antipsychotics was calculated by dividing the total dosage by the duration of the prescription, which was represented as chlorpromazine equivalents,^25^ and categorized into 2 groups: low dose (<200 mg/d) and high dose (≥200 mg/d). The antipsychotic regimen was analyzed based on each prescribed agent or combination of antipsychotic agents: risperidone, aripiprazole, quetiapine, olanzapine, haloperidol, and polypharmacy. Polypharmacy was defined as person-days of overlapped supplying dates of 2 or more antipsychotic agents.

### Outcomes

The primary outcome was the incidence of movement disorders and seizures. The possible movement disorders included parkinsonism, dystonia, extrapyramidal symptoms, chorea, and tic; these were identified through the diagnosis record. Seizure incidence was determined through the diagnosis record traced for prescription of antiepileptics or electroencephalographic examination within 30 days. The *ICD-10* codes for these outcomes are summarized in eTable 3 in the Supplement.

### Potential Confounders

We measured various potential confounding factors that may be associated with the incidence of neurological AEs: sex, age, health insurance, psychiatric comorbidities, psychiatric hospitalization, and comedication with other psychotropic drugs (eTables 4 and 5 in the Supplement). The patient’s age, psychiatric comorbidities, and psychiatric hospitalization (yes or no), and other psychotropic drug use were measured as time-dependent covariates. This approach was used because of the potential associations over time with the risk of developing the outcomes of interest. Patient sex and health insurance type were obtained at the index date.

### Statistical Analysis

Baseline patient demographic characteristics for each cohort were presented as numbers and proportions. Crude incidence rates were calculated as the total number of cases for each exposure per 100 person-years. We developed 2 separate extended Cox proportional hazards models to estimate HRs with 95% CIs for movement disorders and seizures associated with the time-varying exposure of antidepressants and/or antipsychotics. Potential confounders, including sex, age, health insurance type, psychiatric comorbidities, psychiatric hospitalization, and other psychotropic medication use, were adjusted for in these models.

The first assessment was conducted to evaluate the risk of each AE according to nonuse, antidepressant-only use, antipsychotic-only use, and concomitant use of antidepressants and antipsychotics. Second, we investigated how these risks in concomitant use varied by the dose of antipsychotics (low or high) or individual agent, extracting the antidepressants and concomitant use period. For all analyses, the reference exposure was the period of antidepressant-only use.

We also performed sensitivity analyses to account for the uncertainty of exposure, with the duration of the carryover effect being 7 and 28 days. Statistical significance was defined as a 95% CI excluding 1. All statistical analyses were performed using SAS Enterprise Guide, version 6.1 (SAS Institute, Inc).

## Results

**Figure 1.**
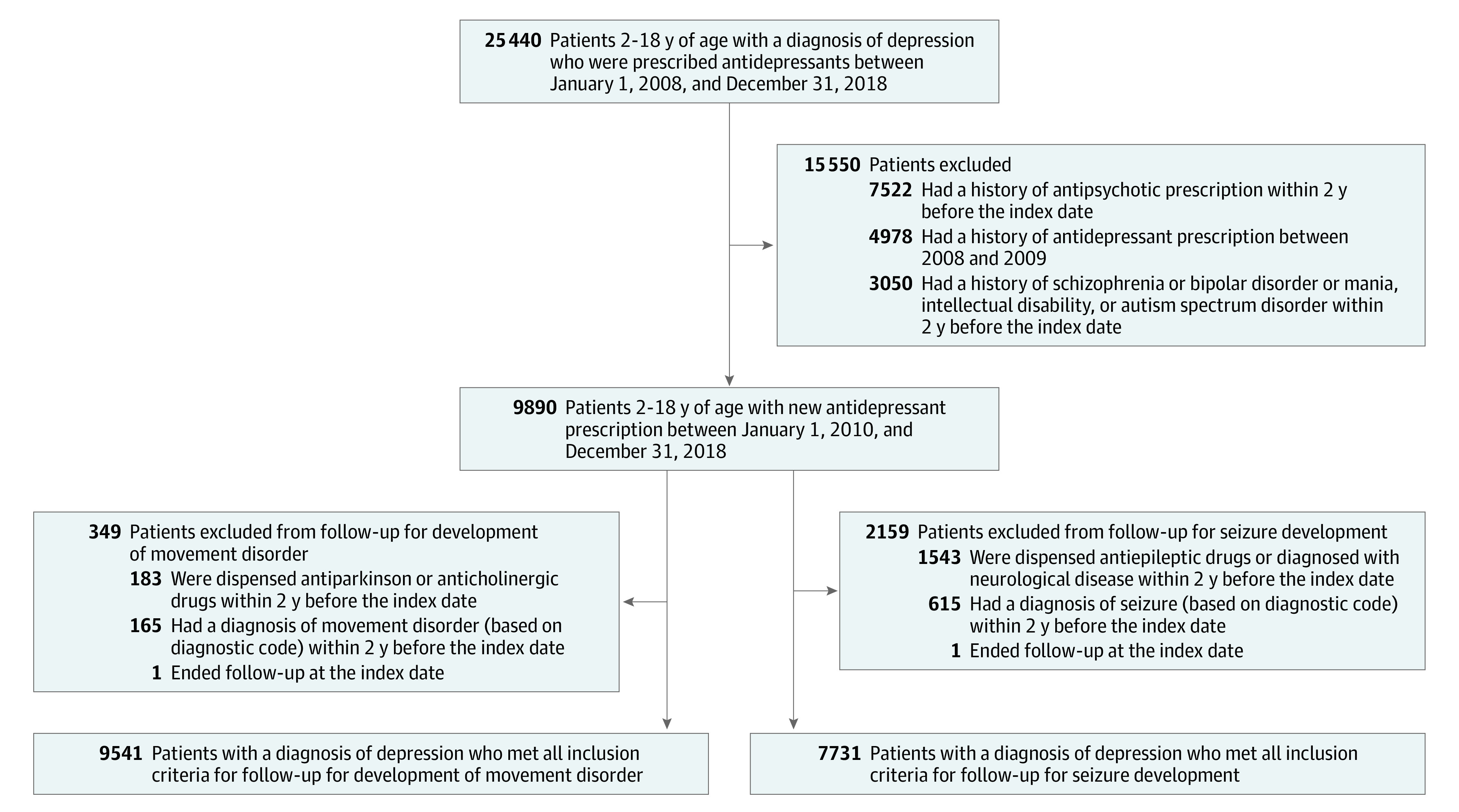
Flowchart of Study Patient Selection and Study Design

There were 9890 participants in the final cohort; among these, 9541 participants (mean [SD] age, 14.8 [2.8] years; 4956 [51.9%] female and 4585 [48.1%] male) were included in the movement disorder cohort and 7731 participants (mean [SD] age, 14.9 [2.7] years; 4150 [53.7%] female and 3581 [46.3%] male) were included in the seizure cohort (Figure 1; Table). Most of the patients were aged 13 to 18 years (7952 [83.4%] in the movement disorder cohort and 6486 [83.9%] in the seizure cohort), and most were within the health insurance group at the index date. Anxiety disorder was the most common mental health disorder listed for 1 year before the index date (1778 [23.0%] in the movement disorder cohort and 2384 [25.0%] in the seizure cohort).

**Table.** Demographic and Clinical Characteristics of the Study Populations in the Movement Disorder and Seizure Cohorts (N = 9890)^a^

Characteristic	No. (%) of patients	
	Movement disorder cohort (n = 9541)	Seizure cohort (n = 7731)
Sex		
Male	4585 (48.1)	3581 (46.3)
Female	4956 (51.9)	4150 (53.7)
Age		
Mean (SD), y	14.8 (2.8)	14.9 (2.7)
2-6	124 (1.3)	86 (1.1)
7-12	1465 (15.4)	1159 (15.0)
13-18	7952 (83.4)	6486 (83.9)
Insurance type		
National Health Insurance Service	8483 (88.9)	6931 (89.7)
Medical Aid	1058 (11.1)	800 (10.4)
Prescription of antipsychotics at the index date		
No	8575 (89.9)	6891 (89.1)
Yes	966 (10.1)	840 (10.9)
Diagnosis of other mental health disorder within 1 y before the index date		
Anxiety disorder	1778 (23.0)	2384 (25.0)
ADHD	1411 (18.3)	1799 (18.9)
Tic disorder	419 (5.4)	545 (5.7)

Abbreviation: ADHD, attention-deficit hyperactivity disorder.

^a^ The movement disorder cohort comprised the 9541 of 9890 patients who met the criteria for follow-up for movement disorders; the seizure cohort comprised the 7731 patients who met the criteria for follow-up for seizure development.

In the movement disorder cohort, there were 1087 incident cases of movement disorders after a mean of 2.70 years of follow-up. The mean follow-up of the seizure cohort was 2.68 years, and there were 722 incident cases of seizure. At the first date of antidepressant treatment, approximately 10% of patients (966 of 9541 patients in the movement disorder cohort and 840 of 7731 in the seizure cohort) concomitantly used antipsychotics. The median duration of the concomitant use period was approximately 21 days in both cohorts.

**Figure 2.**
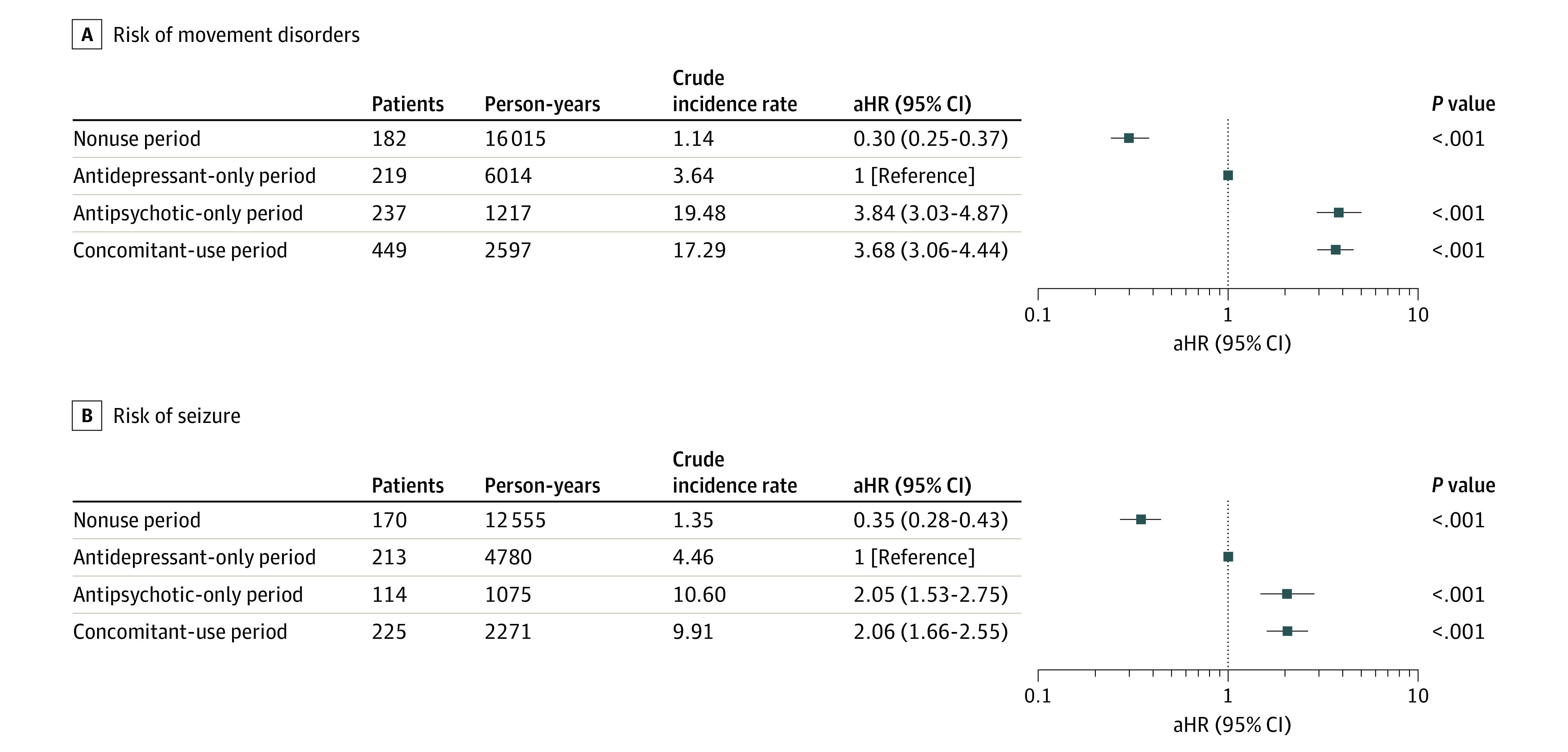
Risk of Movement Disorders and Seizures According to the Exposure to Antipsychotics and/or Antidepressants Hazard ratios were adjusted for sex, age, health insurance type, psychiatric comorbidities, psychiatric hospitalization, and comedication with other psychotropic drugs. To adjust the severity of a psychiatric disorder associated with the occurrence of movement disorders and seizure, inpatient history, mental health diagnosis, and other psychiatric medication use were considered as time-dependent covariates. Crude incidence rates are expressed as the number of cases per 100 person-years. aHR indicates adjusted hazard ratio.

Figure 2 details the risk of movement disorders and seizures related to the time-varying exposure to antidepressants and/or antipsychotics. The crude incidence rates per 100 person-years for movement disorders were 1.14 for nonuse, 3.64 for antidepressant-only use, 19.48 for antipsychotic-only use, and 17.29 for concomitant use. The risk of movement disorders increased in the period of concomitant use (adjusted HR [aHR], 3.68 [95% CI, 3.06-4.44]) and antipsychotic-only use (aHR, 3.84 [95% CI, 3.03-4.87]) compared with antidepressant-only use. According to the exposure status, the crude incidence rate of seizures showed a pattern similar to that of movement disorders (1.35 for nonuse, 4.46 for antidepressant-only use, 10.60 for antipsychotic-only use, and 9.91 for concomitant use per 100 person-years). The aHRs for seizures in the period of concomitant use (2.06; 95% CI, 1.66-2.55) and antipsychotic-only use (2.05; 95% CI, 1.53-2.75) were also greater compared with antidepressant-only use. In both cohorts, the dose of antipsychotics in the concomitant-use period was lower than antipsychotic-only use (eTable 6 in the Supplement).

**Figure 3.**
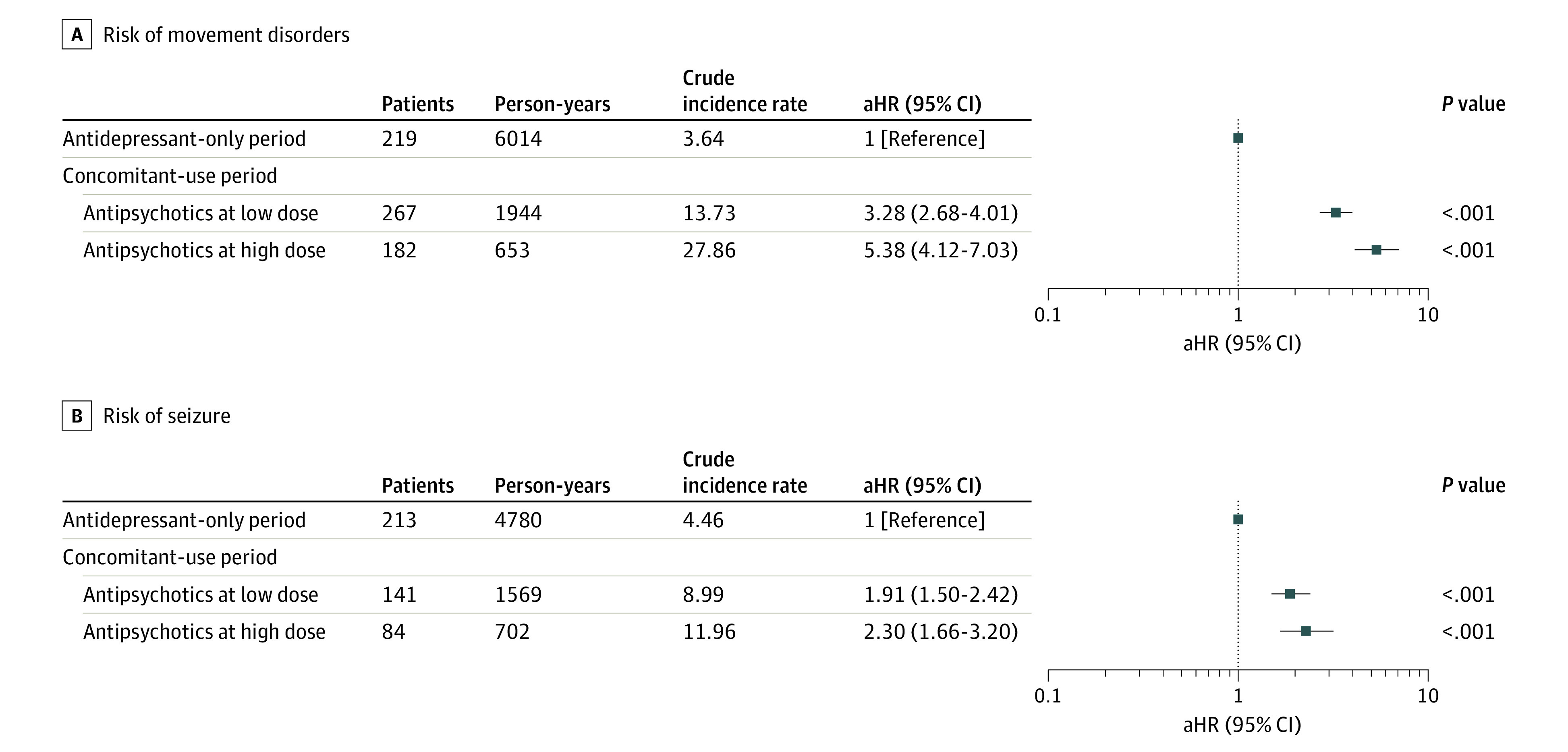
Risk of Movement Disorders or Seizure Incidence According to the Average Daily Dose of Antipsychotics During the Period of Concomitant Use Compared With the Period of Antidepressant Use Hazard ratios were adjusted for sex, age, health insurance type, psychiatric comorbidities, psychiatric hospitalization, and comedication with other psychotropic drugs. To adjust the severity of a psychiatric disorder associated with the occurrence of movement disorders and seizure, inpatient history, mental health diagnosis, and other psychiatric medication use were considered as time-dependent covariates. Crude incidence rates are expressed as the number of cases per 100 person-years. aHR indicates adjusted hazard ratio.

Figure 3 shows the risk of movement disorders or seizures according to the dose of concomitant antipsychotics, with antidepressant use as the reference. An increasing mean daily dose of antipsychotics was associated with an increased risk of movement disorders (low dose: aHR, 3.28 [95% CI, 2.68-4.01]; high dose: aHR, 5.38 [95% CI, 4.12-7.03]). An increasing mean daily dose of antipsychotics was also associated with an increased risk of seizure development (low dose: aHR, 1.91 [95% CI, 1.50-2.42]; high dose: aHR, 2.30 [95% CI, 1.66-3.20]), despite the overlapping CIs between the dose groups.

**Figure 4.**
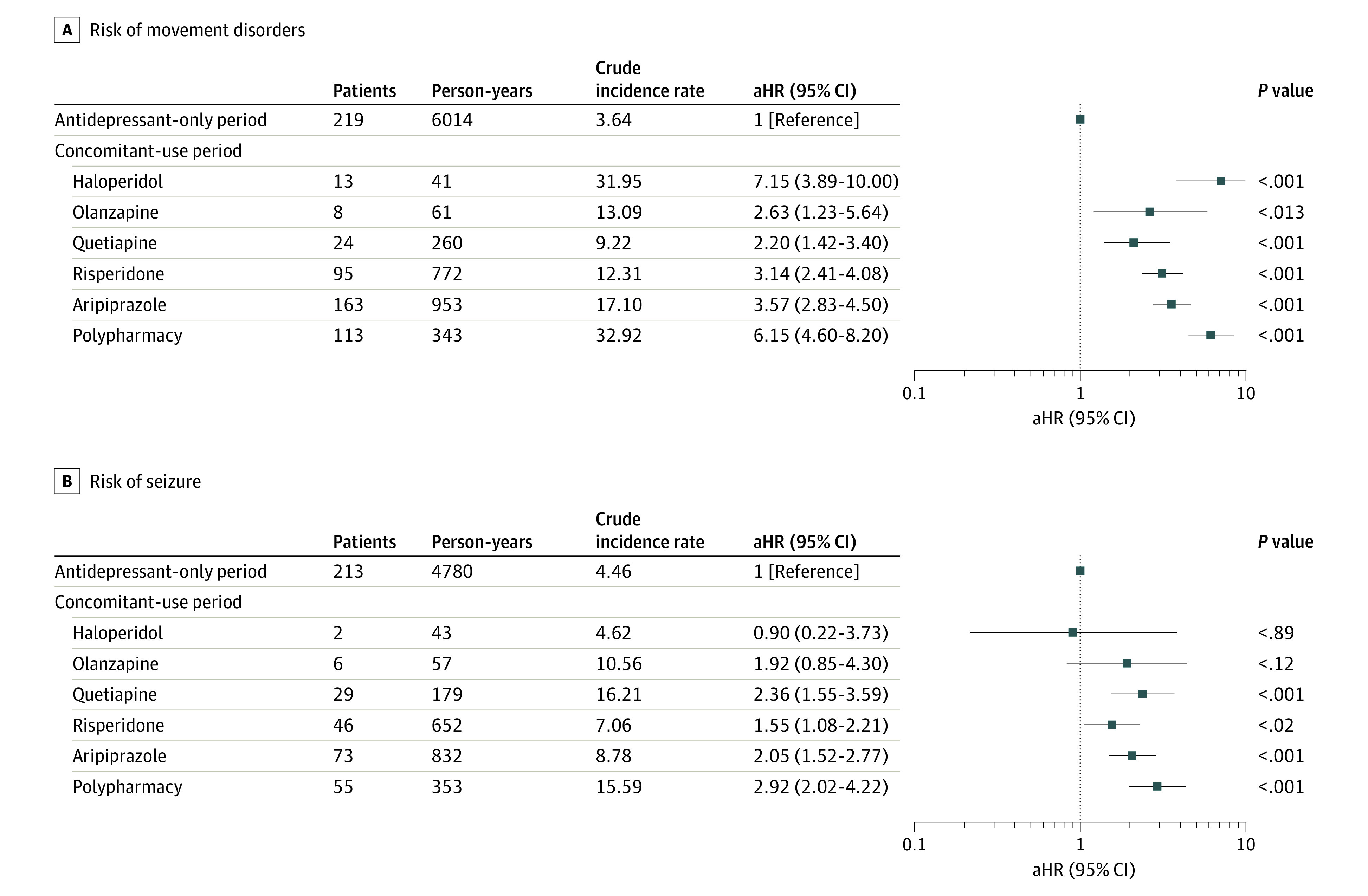
Risk of Movement Disorders or Seizure According to Antipsychotic Agents Used During the Period of Concomitant Use Compared With the Period of Antidepressant-only Use Hazard ratios were adjusted for sex, age, health insurance type, psychiatric comorbidities, psychiatric hospitalization, and comedication with other psychotropic drugs. To adjust the severity of a psychiatric disorder associated with the occurrence of movement disorders and seizure, inpatient history, mental health diagnosis, and other psychiatric medication use were considered as time-dependent covariates. The periods of concomitant use of other antipsychotic agents are not displayed in the figure but were included in the analysis to accurately account for the associations with antipsychotic agents. Polypharmacy was defined as person-days of overlapped supplying dates of more than 2 or more antipsychotic agents. Crude incidence rates are expressed as the number of cases per 100 person-years. aHR indicates adjusted hazard ratio.

The risk of movement disorders or seizures associated with individual antipsychotic agents in the period of concomitant use is presented in Figure 4. The risk of movement disorder was the highest with haloperidol (aHR, 7.15; 95% CI, 3.89-10.00), followed by polypharmacy (aHR, 6.15; 95% CI, 4.60-8.20), aripiprazole (aHR, 3.57; 95% CI, 2.83-4.50), risperidone (aHR, 3.14; 95% CI, 2.41-4.08), olanzapine (aHR, 2.63; 95% CI, 1.23-5.64), and quetiapine (aHR, 2.20; 95% CI, 1.42-3.40). The risk of seizure was highest with polypharmacy (aHR, 2.92; 95% CI, 2.02-4.22), followed by quetiapine (aHR, 2.36; 95% CI, 1.55-3.59), aripiprazole (aHR, 2.05; 95% CI, 1.52-2.77), and risperidone (aHR, 1.55; 95% CI, 1.08-2.21). Among the drugs used concomitantly, the mean (SD) daily doses of aripiprazole and risperidone were 117.52 (66.67) mg/d and 109.11 (64.25) mg/d, respectively. In sensitivity analyses, the associations between neurological AEs and the adjunctive antipsychotics were similar to our primary analysis (eTables 7-12 in the Supplement).

## Discussion

In this cohort study, we observed significant associations between the incidence of movement disorders or seizures and adjunctive treatment with antipsychotics children and adolescents with depression. To our knowledge, this is the first study that evaluated the association of neurological AEs with adjunctive antipsychotic treatment patterns in this patient population.

The results of this study have the following implications. First, using a large sample taken from population-based data, this study provides further evidence of associations between adjunctive antipsychotics and both movement disorders and seizures in children and adolescents with depression. Antipsychotics are increasingly prescribed for a wide range of mental health disorders among children and adolescents, a significant proportion of which are for adjunctive use for depression.^14^ Movement disorders and seizures are representative neurological AEs of antipsychotic use,^26,27^ and antidepressants also independently pose risks of these AEs.^28,29^ Thus, these risks might be further increased when antipsychotics are administered with antidepressants.^29^ In a previous meta-analysis of RCTs^10^ that assessed the efficacy and safety of augmentation with antipsychotics in adults with depression, the discontinuation rate due to AEs was higher (odds ratio 3.91; 95% CI, 2.68-5.72) with adjunctive antipsychotics than with placebo. However, safety data in children and adolescents are still limited,^30^ despite the possible negative effects of these AEs on the neurodevelopment of these patients.^31^ Thus, our study has the advantage of providing information on the different levels of risk of neurological AEs associated with adjuvant antipsychotics in this population.

Second, we identified the exposure of our study medications as time-varying covariates: antidepressant-only use, antipsychotic-only use, concomitant use, and nonuse. This approach could control the immortal time bias,^32^ which has frequently occurred in fixed-time methodology through the misclassification of time points because of the change in exposure status during follow-up.^33,34^ The prescription patterns could be changed over time according to the patient’s symptoms and disease severities.^35,36,37,38,39^ Thus, we might more precisely estimate the risk of movement disorders and seizures associated with the use of antipsychotics and antidepressants in the treatment of children and adolescents with depression.

Third, the period of concomitant use had a higher HR for neurological AEs compared with the period of antidepressant-only use, but it was slightly lower than that for antipsychotic-only use, but the CIs overlapped. One interpretation of this finding is that antipsychotics were associated with increased risks of movement disorders and seizure, but the concomitant use of antipsychotics did not synergize the risk of neurological AEs of antidepressants. Further, the tendency of lower HRs for both AEs during the period of concomitant use compared with antipsychotic-only use might be attributed to the dose-effect of antipsychotics. In our study, the median antipsychotic dose in the period of concomitant use was approximately 40% to 50% lower than in the period of antipsychotic-only use (eTable 12 in the Supplement). However, further studies are needed to elucidate the underlying mechanism of this result.

Nevertheless, attention should be paid to patients who are concomitantly exposed to antidepressants and antipsychotics because of the possibility of undesirable neurological AEs induced by pharmacokinetic drug-drug interactions. Most antipsychotics and antidepressants are metabolized by the cytochrome P450 (CYP) enzyme system, inducing or inhibiting the activation of these enzymes.^40,41,42^ For instance, CYP2D6 is crucial in the metabolism of antipsychotics,^42^ and all serotonin-selective reuptake inhibitors are CYP2D6 inhibitors.^40^ Moreover, CYP enzymes act as substrates, inducers, or inhibitors of P-glycoprotein, which acts as a transporter in drug disposition.^40^ Thus, the plasma concentration of antipsychotics could be increased more than expected, which could induce the development of AEs.

Regardless of which antipsychotic agents were used, HRs for movement disorders were significantly higher with concomitant use compared with antidepressant-only use. The highest HR for movement disorders was found with concomitant haloperidol use; these results are consistent with existing studies, wherein typical antipsychotics, such as haloperidol, conferred a higher risk of movement disorders than atypical antipsychotics.^43,44^ Additionally, the relatively lower HRs for olanzapine and quetiapine use were expected, as they have been shown to have a lower affinity for dopaminergic D2 receptors than others.^45^ A previous meta-analysis reported that among the atypical antipsychotics (quetiapine, risperidone, aripiprazole, and olanzapine), quetiapine was used least often for the treatment of parkinsonism, followed by olanzapine.^46^

We also have provided quantitative evidence of the association between the concomitant use of antidepressants with antipsychotics and seizure risk. During the period of concomitant use, except for polypharmacy, quetiapine had the highest HR for seizures. Olanzapine use was associated with a higher risk of seizures in terms of crude incidence rate than antidepressant-only use, but these results did not hold up in the adjusted analysis. This result observed in olanzapine could be associated with the small sample size (57 person-years). Meanwhile, it is noteworthy that the administration of aripiprazole had a higher HR for seizure than quetiapine and olanzapine. Aripiprazole is widely recognized as having a lower seizure risk than other agents^22,47,48,49^; in the previous studies conducted by our research group, risperidone showed a similar incidence risk of seizures.^22^ Considering the slightly higher dose of aripiprazole than risperidone in the period of concomitant use, this discrepancy with previous findings may be attributed to differences in dosages for each agent. Among the drugs used concomitantly, the mean daily dose of aripiprazole was slightly higher than that of risperidone (risperidone, 109.11 mg/d vs aripiprazole, 117.52 mg/d). Although further research is required, this study provides evidence of risks associated with high-dose antipsychotics, particularly within the pediatric population.

### Limitations

This study has several limitations. First, antipsychotics prescribed for treating comorbidities other than depression could not be separately defined, which could serve as a potential confounder for outcomes. To minimize the confounding effect by comorbidities, we applied the following methods: (1) patients who require antipsychotic treatment for conditions other than depression were excluded from the study cohort; (2) in creating the movement disorder cohort and seizure cohort, patients with any diagnosis record of disorders associated with each outcome for at least 2 years before the index date were excluded; and (3) new diagnoses of psychiatric disorders, psychiatric hospitalization, and comedication (such as antidepressants, antianxiety drugs, and stimulants) prescriptions were identified. We then adjusted these factors as time-varying covariates during the follow-up period. Second, outcome misclassification is possible, as our study was based on the claims database. Third, the possibility of exposure status misclassification may exist because the prescription data from the claims data had no information on patients’ adherence to treatment.

## Conclusions

These results suggest that the use of antipsychotics as either monotherapy or adjuvant with antidepressants is associated with an increased risk of movement disorders and seizures in children and adolescents with depression. During the period of concomitant use, this risk may be dose dependent, with individual antipsychotic agents conferring different levels of risk. These results indicate that health care professionals need to carefully consider the risk-benefit profile of depression treatments in children and adolescents.

## Notes

Supplement. **eTable 1.** The List of Codes for the Diagnosis and Medications Used for Creating the Movement Disorder–Free Cohorts **eTable 2.** The List of Codes for the Diagnosis and Medications Used for Creating the Seizure-Free Cohorts **eTable 3.** The List of Codes for the Diagnosis and Medications Used for Identifying the Incidence of Movement Disorders and Seizure **eTable 4.** *ICD-10* Codes for Psychiatric Disorder Records **eTable 5.** ATC Codes for Other Psychiatric Medications **eTable 6.** Median of Antipsychotics Dose in the Period With Antipsychotic and Concomitant Use **eTable 7.** Risk of Movement Disorders or Seizures According to the Exposure Status to Antipsychotics and/or Antidepressants (the Duration of the Carryover Effect Was Assumed as 7 Days) **eTable 8.** Risk of Movement Disorders or Seizure Incidence According to an Average Daily Dose of Antipsychotics During the Period of Concomitant Use Compared With the Period of Antidepressant Use (the Duration of the Carryover Effect Was Assumed as 7 Days) **eTable 9.** Risk of Movement Disorders or Seizure Incidence According to Antipsychotic Agents During the Period of Concomitant Use Compared With the Period of Antidepressant Use (the Duration of the Carryover Effect Was Assumed as 7 Days) **eTable 10.** Risk of Movement Disorders or Seizures According to the Exposure Status to Antipsychotics and/or Antidepressants (the Duration of the Carryover Effect Was Assumed as 28 Days) **eTable 11.** Risk of Movement Disorders or Seizure Incidence According to an Average Daily Dose of Antipsychotics During the Period of Concomitant Use Compared With the Period of Antidepressant Use (the Duration of the Carryover Effect Was Assumed as 28 Days) **eTable 12.** Risk of Movement Disorders or Seizure Incidence According to Antipsychotic Agents During the Period of Concomitant Use Compared With the Period of Antidepressant Use (the Duration of the Carryover Effect Was Assumed as 28 Days)
