# Supplementary material for: Association of Treatment With Antipsychotics, Antidepressants, or Both With Movement Disorders and Seizures Among Children and Adolescents With Depression in Korea
Source: JAMA Netw Open. 2022 Apr 15;5(4):e227074. doi: 10.1001/jamanetworkopen.2022.7074 (PMC9012964; doi:10.1001/jamanetworkopen.2022.7074)
Supplement: Supplement. — eTable 1. The List of Codes for the Diagnosis and Medications Used for Creating the Movement Disorder–Free Cohorts eTable 2. The List of Codes for the Diagnosis and Medications Used for Creating the Seizure-Free Cohorts eTable 3. The List of Codes for the Diagnosis and Medications Used for Identifying the Incidence of Movement Disorders and Seizure eTable 4. ICD-10 Codes for Psychiatric Disorder Records eTable 5. ATC Codes for Other Psychiatric Medications eTable 6. Median of Antipsychotics Dose in the Period With Antipsychotic and Concomitant Use eTable 7. Risk of Movement Disorders or Seizures According to the Exposure Status to Antipsychotics and/or Antidepressants (the Duration of the Carryover Effect Was Assumed as 7 Days) eTable 8. Risk of Movement Disorders or Seizure Incidence According to an Average Daily Dose of Antipsychotics During the Period of Concomitant Use Compared With the Period of Antidepressant Use (the Duration of the Carryover Effect Was Assumed as 7 Days) eTable 9. Risk of Movement Disorders or Seizure Incidence According to Antipsychotic Agents During the Period of Concomitant Use Compared With the Period of Antidepressant Use (the Duration of the Carryover Effect Was Assumed as 7 Days) eTable 10. Risk of Movement Disorders or Seizures According to the Exposure Status to Antipsychotics and/or Antidepressants (the Duration of the Carryover Effect Was Assumed as 28 Days) eTable 11. Risk of Movement Disorders or Seizure Incidence According to an Average Daily Dose of Antipsychotics During the Period of Concomitant Use Compared With the Period of Antidepressant Use (the Duration of the Carryover Effect Was Assumed as 28 Days) eTable 12. Risk of Movement Disorders or Seizure Incidence According to Antipsychotic Agents During the Period of Concomitant Use Compared With the Period of Antidepressant Use (the Duration of the Carryover Effect Was Assumed as 28 Days) [file jamanetwopen-e227074-s001.pdf]

Supplemental Online Content

Jeon SM, Park HY, Park S, Chung US, Kwon JW. Association of treatment with antipsychotics, antidepressants, or both with movement disorders and seizures among children and adolescents with depression in Korea. *JAMA Netw Open.* 2022;5(4):e227074.
doi:10.1001/jamanetworkopen.2022.7074

eTable 1. The List of Codes for the Diagnosis and Medications Used for Creating the Movement Disorder–Free Cohorts

eTable 2. The List of Codes for the Diagnosis and Medications Used for Creating the Seizure-Free Cohorts

eTable 3. The List of Codes for the Diagnosis and Medications Used for Identifying the Incidence of Movement Disorders and Seizure

eTable 4. *ICD-10* Codes for Psychiatric Disorder Records

eTable 5. ATC Codes for Other Psychiatric Medications

eTable 6. Median of Antipsychotics Dose in the Period With Antipsychotic and Concomitant Use

eTable 7. Risk of Movement Disorders or Seizures According to the Exposure Status to Antipsychotics and/or Antidepressants (the Duration of the Carryover Effect Was Assumed as 7 Days)

eTable 8. Risk of Movement Disorders or Seizure Incidence According to an Average Daily Dose of Antipsychotics During the Period of Concomitant Use Compared With the Period of Antidepressant Use (the Duration of the Carryover Effect Was Assumed as 7 Days)

eTable 9. Risk of Movement Disorders or Seizure Incidence According to Antipsychotic Agents During the Period of Concomitant Use Compared With the Period of Antidepressant Use (the Duration of the Carryover Effect Was Assumed as 7 Days)

eTable 10. Risk of Movement Disorders or Seizures According to the Exposure Status to Antipsychotics and/or Antidepressants (the Duration of the Carryover Effect Was Assumed as 28 Days)

eTable 11. Risk of Movement Disorders or Seizure Incidence According to an Average Daily Dose of Antipsychotics During the Period of Concomitant Use Compared With the Period of Antidepressant Use (the Duration of the Carryover Effect Was Assumed as 28 Days)

eTable 12. Risk of Movement Disorders or Seizure Incidence According to Antipsychotic Agents During the Period of Concomitant Use Compared With the Period of Antidepressant Use (the Duration of the Carryover Effect Was Assumed as 28 Days)

This supplemental material has been provided by the authors to give readers additional information about their work.

© 2022 Jeon SM et al. *JAMA Network Open.*

eTable 1. The list of codes for the diagnosis and medications used for creating the movement disorder-free cohorts

Prevalent cases of movement disorders		
**Classification**	**ICD-10 code**	
Movement disorders	G20, G21, G21.1, G21.2, G21.3, G21.4, G21.8, G21.9, G22, G23, G23.1, G23..2, G23.3, G23.8, G23.9, G24, G24.0, G24.1, G24.2, G24.3, G24.5, G24.8, G24.9, G25, G25.0, G25.1, G25.3, G25.4, G25.5, G25.6, G25.8, G25.9 and G26	
Prescription of medication treated for movement disorders		
**Classification**	**ATC code**	**Agents in the classification**
Antiparkinsonian drugs	N04B	Pergolide, Ropinirole, Selegiline, Levodopa and Decarboxylase inhibitor, Pramipexole, Entacapone, and Rasagiline
Anticholinergic drugs	N04A	Trihexyphenidyl, Biperiden, Procyclidine, and Benztropine
ICD, International Classification of Diseases; ATC, Anatomical Therapeutic Chemical		

© 2022 Jeon SM et al. *JAMA Network Open.*

**eTable 2. The list of code for the diagnosis and medications used for creating the seizure-free cohorts**		
Prevalent cases of movement disorders		
**Classification**	**ICD-10 code**	
Seizure	R56, R56.0, R56.8 and G40	
Prescription of medication treated for seizure		
**Classification**	**ATC code**	**Agents in that classification**
Antiepileptic drugs	N03A	Phenobarbital, Primidone, Phenytoin, Fosphenytoin, Ethosuximide, Clonazepam, Carbamazepine, Oxcarbazepine, Rufinamide, Valproic acid, Vigabatrin, Lamotrigine, Topiramate, Gabapentin, Levetiracetam, Zonisamide, Pregabalin, Stiripentol, Lacosamide
Neurological disorders related to seizure		
**Classification**	**ICD-10 code**	
Cerebral palsy	G80	
Congenital anomalies	Q00	
Brain tumor	C71, C79, D32, D33	
CNS pathology (incl. meningitis, encephalitis, celebrities)	G00-G90	
Cerebrovascular disease	I67, I68	
Cerebral trauma	S01,S02,S06	

ICD, International Classification of Diseases; ATC, Anatomical Therapeutic Chemical

© 2022 Jeon SM et al. *JAMA Network Open.*

eTable 3. The list of code for the diagnosis and medications used for identifying the incidence of movement disorders and seizure		
Movement disorders		
**Classification**	**ICD-10 codes**	
**Movemet disorders**	G20, G21, G21.1, G21.2, G21.8, G21.9, G24, G24.0, G24.2, G25, G25.0, G25.1, G25.4, G25.6, and G25.9	
Seizures		
**Classification**	**ICD-10 codes**	
Seizure	G40 and R56.8	
**Classification**	**ATC code**	**Agents in that classification**
Antiepileptic drugs	N03A	Phenobarbital, Primidone, Phenytoin, Fosphenytoin, Ethosuximide, Clonazepam, Carbamazepine, Oxcarbazepine, Rufinamide, Valproic acid, Vigabatrin, Lamotrigine, Topiramate, Gabapentin, Levetiracetam, Zonisamide, Pregabalin, Stiripentol, Lacosamide

ICD, International Classification of Diseases; ATC, Anatomical Therapeutic Chemical

© 2022 Jeon SM et al. *JAMA Network Open.*

eTable 4. ICD-10 codes for psychiatric disorder records

**Psychiatric disorder**	**ICD-10 code**
Anxiety disorder	F40, F41
ADHD	F90
Intellectual disability	F70–F79
Tic disorder	F95
Bipolar disorder	F31
Schizophrenia spectrum	F20–F29
Autism spectrum disorder	F84

ADHD, attention deficit hyperactivity disorder; ICD-10, International Classification of Diseases

© 2022 Jeon SM et al. *JAMA Network Open.*

eTable 5. ATC codes for other psychiatric medications

Classification	ATC code	Agents in the classification
Anticholinergic drugs	N04A	Trihexyphenidyl, Biperiden, Procyclidine, and Benztropine
Antiepileptic drugs	N03A	Phenobarbital, Primidone, Phenytoin, Fosphenytoin, Ethosuximide, Clonazepam, Carbamazepine, Oxcarbazepine, Rufinamide, Valproic acid, Vigabatrin, Lamotrigine, Topiramate, Gabapentin, Levetiracetam, Zonisamide, Pregabalin, Stiripentol, and Lacosamide
Antianxiety drugs	N05B	Diazepam, Chlordiazepoxide, Lorazepam, Bromazepam, Clobazam, Alprazolam, Ethyl loflazepate, Etizolam, Clotiazepam, Tofisopam, Hydroxyzine, and Buspirione
Stimulants for ADHD	N06B	Methylphenidate
Nonstimulants for ADHD	N06B	Modafinil, Atomoxetine, Clonidine

ADHD, attention deficit hyperactivity disorder

© 2022 Jeon SM et al. *JAMA Network Open.*

eTable 6. Median of antipsychotics dose in the period with antipsychotic and concomitant use

	Antipsychotic-only use	Concomitant use
Movement disorders-free cohort		
All patients	158.57	93.33
2–6	87.61	66.67
7–12	75.00	65.04
13–18	181.92	99.11
Seizure-free cohort		
All patients	200	100
2–6	88.47	64.17
7–12	80.47	66.67
13–18	217.05	104.00

* The average daily dose (mg/day) was calculated as a chlorpromazine-equivalent dose

© 2022 Jeon SM et al. *JAMA Network Open.*

**eTable 7. Risk of movement disorders or seizures according to the exposure status to antipsychotics and/or antidepressants (the duration of the carry-over effect was assumed as 7 days)**				
	**Cases**	**Person-years**	**Crude Incidence rate[a]**	**Adjusted HR (95% CI)[b]**
Movement disorders				
Nonuse period	245	16,613	1.47	0.383 (0.316–0.463)
Antidepressant use period	213	5,640	3.78	1.00 (Reference)
Antipsychotics use period	234	1,197	19.55	3.893 (3.067–4.941)
Concomitant use period	395	2,393	16.51	3.459 (2.855–4.191)
Seizure				
Nonuse period	218	13,029	1.67	0.426 (0.349–0.52)
Antidepressant use period	203	4,492	4.52	1.00 (Reference)
Antipsychotics use period	108	1,056	10.22	1.834 (1.353–2.486)
Concomitant use period	193	2,103	9.18	1.847 (1.472–2.318)
HR, Hazard ratio; CI, Confidence intervals				

[a]Crude incidence rate are expressed as the number of cases per 100 person-years
[b]Adjusted for sex, age, insurance type, inpatient history, and psychiatric diagnosis. To consider the severity of a psychiatric disorder related to the occurrence of movement disorders and seizure, covariates of inpatient history, mental health diagnosis, and other psychiatric medication use considered as time-dependent were used in this model.

© 2022 Jeon SM et al. *JAMA Network Open.*

**eTable 8. Risk of movement disorders or seizure incidence according to an average daily dose of antipsychotics during the period of concomitant use compared with the period of antidepressant use (the duration of the carry-over effect was assumed as 7 days)**				
	**Cases**	**Person-years**	**Crude Incidence rate[a]**	**Adjusted HR (95% CI)[b]**
Movement disorders				
Antidepressant-only use period	213	5,640	3.78	1.00 (Reference)
Dose of antipsychotics during the concomitant use period[b]				
Antipsychotics at low dose	268	1,925	13.92	3.27 (2.669–4.008)
Antipsychotics at high dose	127	468	27.12	5.113 (3.79–6.899)
Seizure				
Antidepressant-only use period	203	4,492	4.52	1.00 (Reference)
Dose of antipsychotics during the concomitant use period[b]				
Antipsychotics at low dose	134	1,576	8.50	1.748 (1.359–2.248)
Antipsychotics at high dose	59	527	11.20	2.033 (1.413–2.925)

HR = Hazard ratios; CI = Confidence intervals

[a]Crude incidence rate are expressed as the number of cases per 100 person-years
[b]Adjusted for sex, age, insurance type, inpatient history, and psychiatric diagnosis. To consider the severity of a psychiatric disorder related to the occurrence of movement disorders and seizure, covariates of inpatient history, mental health diagnosis, and other psychiatric medication use considered as time-dependent were used in this model.
[c]Average daily dose were calculated as chlorpromazine equivalent dose and categorized into two groups as follows: Low dose < 200 mg/day and High dose ≥ 200 mg/day

© 2022 Jeon SM et al. *JAMA Network Open.*

eTable 9. Risk of movement disorders or seizure incidence according to antipsychotic agents during the period of concomitant use compared with the period of antidepressant use (the duration of the carry-over effect was assumed as 7 days)

	Cases	Person-years	Crude Incidence rate[a]	Adjusted HR (95% CI)[b]
Movement disorder				
Antidepressant-only use period	213	5,640	3.78	1.00 (Reference)
Concomitant use period				
Haloperidol	13	37	35.30	7.529 (4.078–13.899)
Olanzapine	6	59	10.17	2.036 (0.864–4.797)
Quetiapine	26	243	10.70	2.449 (1.602–3.745)
Risperidone	92	723	12.72	3.263 (2.49–4.275)
Aripiprazole	150	893	16.80	3.474 (2.739–4.405)
Other typical antipsychotics	12	59	20.44	5.415 (2.948–9.947)
Other atypical antipsychotics	18	97	18.55	3.802 (2.224–6.501)
Polypharmacy[c]	78	282	27.64	5.019 (3.62–6.96)
Seizure				
Antidepressant-only use period	203	4,492	4.52	1.00 (Reference)
Concomitant use period				
Haloperidol	2	40	5.00	0.964 (0.234–3.981)
Olanzapine	6	55	10.88	1.871 (0.832–4.205)
Quetiapine	25	169	14.79	2.07 (1.322–3.242)
Risperidone	40	614	6.52	1.376 (0.944–2.006)
Aripiprazole	64	781	8.20	1.844 (1.338–2.54)
Other typical antipsychotics	7	46	15.27	2.518 (1.216–5.215)
Other atypical antipsychotics	8	101	7.95	1.57 (0.759–3.248)
Polypharmacy[c]	41	298	13.75	2.417 (1.596–3.66)

HR, Hazard ratios; CI, Confidence intervals

[a]Crude incidence rate are expressed as the number of cases per 100 person-years

[b]Adjusted for sex, age, insurance type, inpatient history, and psychiatric diagnosis. To consider the severity of a psychiatric disorder related to the occurrence of movement disorders and seizure, covariates of inpatient history, mental health diagnosis, and other psychiatric medication use considered as time-dependent were used in this model.

[c]Polypharmacy was defined as the person-day of overlapped supplying dates of more than two antipsychotic agents.

© 2022 Jeon SM et al. *JAMA Network Open.*

eTable 10. Risk of movement disorders or seizures according to the exposure status to antipsychotics and/or antidepressants (the duration of the carry-over effect was assumed as 28 days)

	Cases	Person-years	Crude Incidence rate[a]	Adjusted HR (95% CI)[b]
Movement disorders				
Nonuse period	145	15,255	0.95	0.281 (0.225–0.35)
Antidepressant use period	209	6,460	3.24	1.00 (Reference)
Antipsychotics use period	221	1,221	18.09	3.857 (3.047–4.882)
Concomitant use period	512	2,906	17.62	4.177 (3.476–5.019)
Seizure				
Nonuse period	132	11,971	1.10	0.304 (0.243–0.379)
Antidepressant use period	213	5,104	4.17	1.00 (Reference)
Antipsychotics use period	113	1,080	10.47	2.176 (1.614–2.933)
Concomitant use period	264	2,527	10.45	2.282 (1.861–2.799)

HR, Hazard ratio; CI, Confidence intervals

aCrude incidence rate are expressed as the number of cases per 100 person-years
bAdjusted for sex, age, insurance type, inpatient history, and psychiatric diagnosis. To consider the severity of a psychiatric disorder related to the occurrence of movement disorders and seizure, covariates of inpatient history, mental health diagnosis, and other psychiatric medication use considered as time-dependent were used in this model.

© 2022 Jeon SM et al. *JAMA Network Open.*

eTable 11. Risk of movement disorders or seizure incidence according to an average daily dose of antipsychotics during the period of concomitant use compared with the period of antidepressant use (the duration of the carry-over effect was assumed as 28 days)

	**Cases**	**Person-years**	**Crude Incidence rate[a]**	**Adjusted HR (95% CI)[b]**
Movement disorders-free cohort				
Antidepressant-only use period	209	6,460	3.24	1.00 (Reference)
Dose of antipsychotics during the concomitant use period[b]				
Antipsychotics at low dose	262	1,943	13.48	3.644 (2.985–4.448)
Antipsychotics at high dose	250	963	25.96	5.616 (4.418–7.139)
Seizure-free cohort				
Antidepressant-only use period	213	5,104	4.17	1.00 (Reference)
Dose of antipsychotics during the concomitant use period[b]				
Antipsychotics at low dose	146	1,531	9.53	2.118 (1.684–2.665)
Antipsychotics at high dose	118	995	11.85	2.148 (1.603–2.879)

HR = Hazard ratios; CI = Confidence intervals

[a]Crude incidence rate are expressed as the number of cases per 100 person-years

[b]Adjusted for sex, age, insurance type, inpatient history, and psychiatric diagnosis. To consider the severity of a psychiatric disorder related to the occurrence of movement disorders and seizure, covariates of inpatient history, mental health diagnosis, and other psychiatric medication use considered as time-dependent were used in this model.

[c]Average daily dose were calculated as chlorpromazine equivalent dose and categorized into two groups as follows: Low dose < 200 mg/day and High dose ≥ 200 mg/day

© 2022 Jeon SM et al. *JAMA Network Open.*

eTable 12. Risk of movement disorders or seizure incidence according to antipsychotic agents during the period of concomitant use compared with the period of antidepressant use (the duration of the carry-over effect was assumed as 28 days)

	Cases	Person-years	Crude Incidence rate[a]	Adjusted HR (95% CI)[b]
Movement disorder-free cohort				
Antidepressants use period	209	6,460	3.24	1.00 (Reference)
Concomitant use period				
Haloperidol	14	46	30.15	7.491 (4.165–13.473)
Olanzapine	10	64	15.69	3.552 (1.816–6.948)
Quetiapine	26	282	9.21	2.444 (1.604–3.723)
Risperidone	109	840	12.98	3.72 (2.884–4.797)
Aripiprazole	167	1,033	16.16	3.819 (3.044–4.79)
Other typical antipsychotics	14	76	18.52	5.636 (3.2–9.927)
Other atypical antipsychotics	21	106	19.81	4.614 (2.808–7.581)
Polypharmacy[c]	151	459	32.87	6.92 (5.327–8.99)
Seizure-free cohort				
Antidepressants use	213	5,104	4.17	1.00 (Reference)
Concomitant use period				
Haloperidol	1	48	2.08	0.393 (0.054–2.837)
Olanzapine	6	59	10.24	1.974 (0.829–4.704)
Quetiapine	34	192	17.70	2.705 (1.83–3.998)
Risperidone	52	703	7.40	1.647 (1.171–2.318)
Aripiprazole	77	900	8.55	2.134 (1.596–2.853)
Other typical antipsychotics	6	59	10.11	1.743 (0.803–3.782)
Other atypical antipsychotics	10	109	9.17	1.94 (1.032–3.644)
Polypharmacy[c]	78	457	17.08	3.088 (2.244–4.248)

HR, Hazard ratios; CI, Confidence intervals

[a]Crude incidence rate are expressed as the number of cases per 100 person-years
[b]Adjusted for sex, age, insurance type, inpatient history, and psychiatric diagnosis. To consider the severity of a psychiatric disorder related to the occurrence of movement disorders and seizure, covariates of inpatient history, mental health diagnosis, and other psychiatric medication use considered as time-dependent were used in this model.
[c]Polypharmacy was defined as the person-day of overlapped supplying dates of more than two antipsychotic agents.

© 2022 Jeon SM et al. *JAMA Network Open.*
